# Screening for Methicillin-resistant *Staphylococcus aureus* in a residence home for elderly in Germany

**DOI:** 10.1186/s12995-017-0149-6

**Published:** 2017-02-03

**Authors:** Jürgen Becker, Roland Diel

**Affiliations:** 1 Cepheid GmbH, Unterlindau 29, 60323 Frankfurt, Germany; 2 Institute for Epidemiology, University Medical Hospital Schleswig-Holstein, Kiel, Germany

**Keywords:** MRSA, Prevalence, Screening, Feasibility, Nursing home, health care workers

## Abstract

**Background:**

Since many hospitals report high MRSA colonization rates among elderly patients, and because it has been shown that *S. aureus* colonization increases with advancing age, there are concerns about the introduction of MRSA into nursing homes by MRSA positive patients discharged from hospital. So far, admission screening and subsequent longitudinal screening in residence homes or screening at time of hospital discharge is not established on a regular base. On the other hand, MRSA is acquired frequently during hospital stay. Therefore, the MRSA status of residents remains unclear at the time of re-admission to the residence home. This study was conducted to evaluate the rate of nasal MRSA carriage among residents and nursing staffs of 2 nursing homes for the elderly, the potential acquisition of MRSA during a hospital stay and the feasibility to perform direct screening tests in nursing homes for elderly.

**Methods:**

In a study period of 5 months, possibility of active PCR-based screening for MRSA has been tested within 2 residence homes for the elderly, with the obligation to avoid inconvenience to the daily working time and working schedule. Residents and staff members were included in the study and positive test results were confirmed with MRSA culture.

**Results:**

Feasibility of active on site screening in a residence home for the elderly using a rapid PCR method has been confirmed. 154 of 156 residents participated on baseline testing for all current and new admitted residents. In 9 participating residents with former unknown status, nasal carriage with MRSA was confirmed (5.8%). Among 32 documented and eligible movements between the nursing home and the hospital, MRSA could be confirmed after return to the residence home in 2 cases (6.3%). MRSA could also be detected in 1 of 14 participating nursing staff (7.1%).

**Conclusion:**

Prevalence of MRSA was in a range that has been observed for nursing homes in Germany in previous studies. Residents can acquire MRSA during a hospital stay so that further spread after re-admission into the nursing home cannot be excluded. This study shows that easy to perform direct screening tests in outpatient facilities for nursing of the elderly are promising tools as part of potential new strategies for transmission and infection control in such facilities. Additional studies are needed to investigate if screening followed by interventional hygiene measures can reduce MRSA transmission and infection in such facilities.

## Background

Increased life expectancy and demographic changes have led to an increase in the proportion of older people who require medical care in hospitals. In addition, more and more patients are released early after in-patient hospital care, but then require care in outpatient facilities for geriatric nursing or residence homes [1]. Since many hospitals report high MRSA colonization rates among elderly patients, and because it has been shown that *S. aureus* colonization increases with advancing age, there are concerns about the introduction of MRSA into nursing homes by MRSA positive patients discharged from hospital. It is likely that the prevalence of MRSA within nursing homes is increasing as a result of the increased prevalence of MRSA within hospitals, which may have been compounded by the considerable movement of patients from long-stay hospitals to community-based nursing homes [2]. Once introduced, the subsequent spread of MRSA between patients would create a reservoir of MRSA within a nursing home [3], providing the potential for an outbreak and further hospital outbreaks when affected nursing home residents require hospital treatment [2]. Nursing homes provide an ideal environment for the acquisition and spread of MRSA, since residents have an increased risk of colonization due to known risk factors [4–9]. Notably risk factors for pre-existing colonization and new acquisition within nursing facilities can be different [8]. MRSA colonization is also a marker of mortality risk amongst nursing home residents [10, 11].

Additionally, it has been observed that Health Care Workers (HCW) have an increased risk for colonization and contribute to the transmission of MRSA and subsequent infections in hospitals and residence homes [12–17].

In Germany, patients who are at risk for colonization with MRSA are screened at the time of hospital admission including elderly people from geriatric nursing facilities and residence homes. Screening at time of discharge from the hospital is not performed on a regular basis. This is important because patients with a negative screening result at time of admission can colonize during the subsequent hospital stay [18–22]. Based on this, future strategies for the prevention of MRSA transmission and MRSA infection potentially need to include admission screening and subsequent longitudinal screening in residence homes and other outpatient facilities. This study was conducted to evaluate the rate of nasal MRSA carriage among residents and nursing staff of two nursing homes for elderly, the potential risk of MRSA-acquisition for the residents during a hospital stay and the feasibility to perform direct screening tests in nursing homes for the elderly as part of potential new strategies for transmission and infection control in such facilities.

## Methods

### Study sites

The study was conducted in 2 residence homes for elderly located in the federal state of Baden-Württemberg, Germany. Both facilities had a mixed but similar structure of residents including residents with normal health status and the ability to participate in social life and activities within the facility and those with chronic diseases like renal impairment, diabetes, incontinence with the need for urinary tract catheters and others. During the study period, both facilities together provided 138 places for long term care and additional places for short term and single day care. Including new admissions and deaths, 154 residents lived in the 2 residence homes during the study period of which 152 participated on the study.

### Study period

The first specimen was collected on 18th of February 2013. The last specimen was processed on 27th of July 2013.

### MRSA testing assay

All measurements were performed directly in the facilities using the Xpert® MRSA/SA nasal complete test on the Gene Xpert® device platform (Cepheid GmbH, Frankfurt a. M., Germany). The test allows rapid point-of-care testing for MRSA on nasal specimen swabs collected from the residents and nursing staff. The test also detects *S. aureus* without genetic markers for methicillin resistance (MSSA) at the same time. Here, data for MSSA were not reported and discussed because there was no relation to the aim of this study. The test has a sensitivity and specificity of 88.2 and 98.3% compared to enrichment broth with subsequent differentiation [23]. A negative testing result predicts the absence of a positive MRSA culture by 98.0% if the prevalence is 14.0% [23]. All persons gave written consent before participation after adequate explanation. An ethical vote was not necessary because only data in the scope of routine hygiene prevention were recorded.

### Collection and processing of specimen

Collection of nasal specimens was performed according to manufactures recommendation in the package insert using a dual swab validated for the test and provided from the manufacturer. The dual swab was introduced in both nares and turned around carefully one fold with slight pressure. Swabs with nasal specimen were processed according to the manufacturer’s description in the package insert. The test was performed as point-of-care test in the residence home in line with the definitions for point-of-care testing in Germany, as provided in the Guidelines of the German Medical Association on Quality Assurance in Medical Laboratory Testing (http://www.bundesaertzekammer.de). One tip of the dual swab was placed into the vial and broken off at the stem. The vial contains 2 ml of an elution reagent and was delivered from the manufacturer. The vial was closed with the cap and mixed at high speed for 10 s using a vortex mixer. After this, the elution reagent was transferred into the test cartridge using a sterile transfer pipette followed by starting the test on the device. The second tip of the dual swab remained in the original transport container for culture confirmation in an external laboratory if the PCR testing result was positive for MRSA

### Storage of specimen

Swabs used for specimen collection during the day were placed into the plastic transport tube and stored at 6 degrees Celsius until processing for MRSA testing. Testing procedure was done either in the afternoon on the same day or on the next day at morning.

### Measuring regime

Baseline evaluation of the MRSA colonization rate was performed on all residents already living in the 2 facilities who were willing to participate in the study. After the baseline evaluation, screening was focused on newly admitted residents and residents transferred to the hospital at the time of return from the hospital or 1 day later. No measurements were performed in residents at time of transfer from the facility to a hospital. Fourteen of about 20 nursing staff also volunteered to be tested for MRSA.

### Education and assignment

In each facility, collection and processing of the specimens for MRSA testing was assigned to 2 dedicated employees. The employees received a 3 h education in specimen collection and the assay from an authorized technician of the manufacturer. In addition, the first tests on participants of the study were supervised by the technician. In order to investigate the technical and organizational feasibility without inconvenience against the daily working routine and working time, the dedicated employees decided for each day and on their own how much specimen they can collect on the individual day and if sample processing for testing can be performed on the same day or must be scheduled for the day after. These employees had directive function in the institution allowing them to adapt working time and schedule to collect the specimen and perform the tests. During the study period, assigned employees arranged their long term holidays in such a way that one of them was always available at the facility.

### Inclusion and exclusion criteria

Only residents and staff members who were 18 years or older and willing to participate in the study were included. Residents and staff members with injuries of the nasal mucosa were excluded.

### Data collection from hospitals

For moving patients who were positive for MRSA at time of re-admission after hospital stay, results for admission screening in the hospital were collected to allow differentiation if acquisition took place in the hospital or if the patient newly acquired MRSA in the resident home or potentially during transfer to the hospital.

### Culture confirmation

To avoid mis-classification, the second swab was sent to a laboratory for culture confirmation if the PCR-based testing result was positive because a positive result can appear due to former intermittent colonization. Based on the low MRSA prevalence in Germany, the positive predictive value is weak (60.0 – 80.0% or less) making culture confirmation essential and in addition, testing for successful de-colonization is not possible using PCR [6]. Negative PCR testing results were not confirmed via culture due to a high negative predictive value. This is in line with common practice in Germany and underlying recommendations for screening of asymptomatic carriers [6].

### Statistical methods

Microsoft Excel 2010, Version 14.0.4760.10000 was used for calculating the average age, median of age, standard deviation of the age for the entire population and 95% interval of confidence for the average age. Association between the rate of colonization with MRSA and the affiliation to one of the facilities was determined using Fisher’s exact test.

### Transmission control measures

The physician responsible for a resident with a positive MRSA status was informed in order to decide the need of a de-colonization procedure. Transmission control measures for MRSA positive patients were conducted according to the internal standards.

### Documentation of times

The time needed for specimen collection and assay testing was measured by the designed employees. This included the time needed for the procedure alone and additional time that could be attributed to the procedures, i.e. walking time within the facility to visit the participant for specimen collection. Average time needed per test and per specimen collection procedure was calculated based on the total time at the end of the study.

## Results

### Results of baseline evaluation and new admissions

Baseline measurements including new admissions during the study period were performed on 102 of 102 residents in facility 1 (100%) and 52 of 54 residents in facility 2 (96.3%). Baseline characteristics of participating residents are displayed in Table 1. Distribution of women and men was not significantly different between the 2 facilities at the time of baseline measurement (*p* = 0.69). Participating residents of facility 1 had an average age of 85.8 years (95% CI; 84.4 – 87.1) which was significantly higher (*p* = 0.03) compared to the average age of participating residents from facility 2 (82.5; 95% CI; 79.9 – 85.1).

**Table 1:** Baseline characteristics of participating residents

Facility	Screening rate	Age [years]			Gender distribution	
		Mean	Median	Range	Male N (%)	Female N (%)
1	100.0% (102 of 102)	85.8	86.0	59 – 102	36 (35.3%)	66 (64.7%)
2	96.3% (52 of 54)	82.5	84.0	57 – 104	20 (38.5%)	32 (61.3%)
Total	98.7% (154 of 156)	84.7	85.0	57 – 104	56 (36.4%)	98 (63.6%)

Taking both facilities together, 9 out of 154 residents were positive for MRSA, resulting in a colonization rate of 5.84% (see Table 2). There was no association between the rate of colonization with MRSA (*p* = 0.49) and the affiliation to either of the facilities using Fisher’s exact test. There was no significant difference between the age of residents colonized with MRSA and residents not colonized with MRSA (*p* = 0.47).

**Table 2:** Colonization rate of participating residents at baseline and new admitted residents

Facility	MRSA-negative		MRSA-positive	
	Rate N (%)	Mean age [years] (± SD)	Rate N (%)	Mean age [years]
1	97 (95.1%)	83.0 (±7.9)	5 (4.9%)	85.6
2	48 (92.3%)	84.0 (±8.6)	4 (7.7%)	87.0
Total	145 (94.2%)	83.4 (±8.2)	9 (5.8%)	86.2

*SD* Standard deviation; *N* number

### Results of testing health care workers

One out of 14 nursing staff who participated on the study was positive for MRSA (7.1%). This employee was released from work until successful decolonization was confirmed via three negative culture results.

### Results in moving residents

**Fig. 1:**
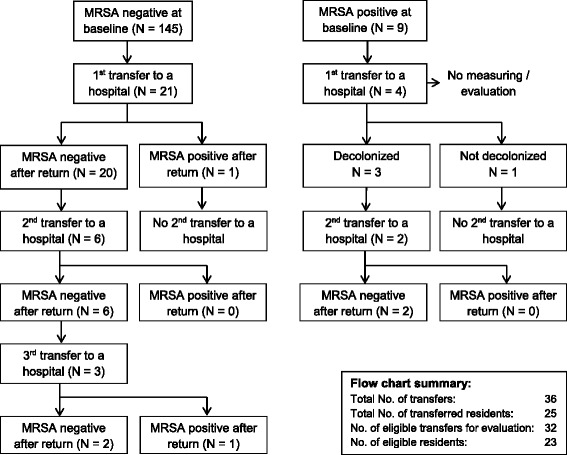
Flow chart of transfers to a hospital and returns to the residence home including results about the MRSA status

During the study period, 21 residents negative for MRSA at baseline were transferred into the hospital due to medical reasons (see Fig. 1). 20 of them were still MRSA negative in the nares after return from the hospital to the residence home. One resident was measured positive for MRSA after return. Average time of hospital stay for the 21 residents was three days. Six of the 20 residents negative for MRSA after the first hospital stay had a second hospital stay. All of them remained negative for MRSA after return to the residence home. Length of stay for the second hospital stay of these 6 residents was 1, 3, 4, 12, 18 and 40 days, respectively. Three of the 6 residents who were negative for MRSA after the second hospital stay had a third hospital stay. Two of them remained negative after return to the resident home and one tested positive for MRSA.

Four residents with positive MRSA status at baseline evaluation were also transferred into the hospital due to medical reasons so that it was possible to inform the hospitals about the history of MRSA colonization or ongoing decolonization procedure. Three of them could be decolonized successfully and one not. The first transfers to the hospital of these four residents were not evaluated concerning potential MRSA acquisition during hospital stay due to ongoing decolonization procedures. Two of these residents with successful, culture proved decolonization had a second hospital stay. Both of these were still negative for MRSA after return from the hospital. Length of stay for these two residents was 14 and 41 days, respectively.

In summary, 25 residents were transferred 1, 2 or 3 times to a hospital and returned back. Of these 25 residents, 17 had 1 transfer (*n* = 17), 5 had 2 transfers (*n* = 10) and 3 had 3 transfers (*n* = 9), resulting in a total amount of 36 transfers. Four transfers and two residents were not eligible for evaluation, resulting in a total sum of 32 transfers and 23 residents who were eligible for data evaluation. In 2 of 32 transfers (6.3%) or 2 of 23 moving residents (8.7%), MRSA could be detected after return from the hospital, respectively.

### Feasibility

In both facilities, sample collection and processing with the test could be integrated into the daily working routine without exceeding the working times. Inconvenience against the working routine was not been reported. Baseline evaluation covering all current residents counted for 84 testing procedures in facility 1 and was finished within 14 working days and 6 tests per day. In facility 2, baseline evaluation counted for 44 testing procedures and was finished within 11 days and 4 tests per day.

After the 128 testing procedures conducted for baseline evaluation at the beginning of the study period, an additional 58 tests have been conducted in the subsequent 5 month period. 26 of these have been conducted on newly admitted residents and 32 on residents after return from the hospital to evaluate colonization status. This means that on average 1 testing procedure per 2.6 days has been conducted when Saturdays and Sundays are included or 1 testing procedure per 1,9 days if the 2 weekend days are excluded.

Including all time consuming steps of the testing procedure, the average time needed for 1 test was 4 min and 18 s. This includes time for manual procedures until testing was started and walking time to the testing device. The maximum time needed in one case was 9 min. Average time needed for specimen collection was 3 min and 27 s per specimen, including walking time within the facility and collection procedure. Maximum time measured for the collection of a specimen in one case was 11 min.

### Assay performance

One hundred eighty six testing procedures were performed of which two measurements (1.1%) needed to be repeated due to invalid testing results. All testing results positive for MRSA could be confirmed via culture as living MRSA organisms.

### Side effects

No side effects in terms of allergic reactions or injuries of the mucosa caused by the specimen collection procedure were observed and reported from either the staff or the residents.

## Discussion

The development and spread of drug resistant bacteria such as MRSA is a threat for the aging populations that have appeared in the past 20 years. Infections caused by MSSA occur more frequently than infections caused by MRSA, but the latter is associated with significantly higher mortality (Odds Ratio: 1.9, 95% CI; 1.5 – 2.4; *p* < 0,001), morbidity and costs [24].

During the 5-month period of our study, baseline evaluation including subsequent new admissions resulted in an MRSA-Prevalence of 5.8% (*N* = 9). This is in line with prevalence data found in other studies from German resident homes for the elderly in which MRSA rates between 1.1 – 26.0% (average of 7.5%) has been reported, suggesting strong facility specific variations as a result of differences in the distribution of risk factors for MRSA colonization and infection [25–33]. None of the nine detected MRSA-positive residents had been identified or known as residents with a history of MRSA at the time of testing indicating that routine screening of residents can detect unknown carriers in such facilities. This supports further discussions about the need for intensified screening of residents to reduce the MRSA rate via transmission control measures. As length of stay in resident homes is normally encompassing the residual life time, MRSA transmission within resident homes is not rare [7, 8]. In a recently published study, new acquired MRSA colonization within a nursing facility was 68.0% related to all colonized residents [8].

In 2 of the 32 eligible transfers (6.3%) involving 23 residents, MRSA could be detected after the return from the hospital. In these cases, acquisition of MRSA during the hospital stay was probably due to a negative screening result at time of hospital admission. In Germany, people from residence homes are defined as patients at risk for MRSA colonization and screened at time of admission to the hospital [6], but screening at time of discharge is not established. On the other hand, colonization with MRSA can occur during hospital stay as reported in relevant studies involving different patient groups and wards [18–22, 34]. Because screening at time of discharge from the hospital is not performed on a regular basis in Germany, MRSA status of these patients remains unknown at time of re-admission into a residence home or another out-patient facility. In addition, data from a recently published nationwide cross-sectional study in residential geriatric nursing facilities in Germany showed that, with local differences, only 41.0 – 71.0% of the participating facilities are provided with information about the presence of multidrug resistant organisms when a resident is transferred (or returned) from hospital to residential geriatric nursing [35]. In combination with the results of our study, this supports further discussions about the need of admission screening in such facilities, or the need for screening of MRSA at time of hospital discharge to avoid MRSA infection in subsequent outpatient facilities.

Of the 14 participating nursing staff, 1 (7.1%) has been found to be positive. A colonization rate of 11.0% for nursing staff in residence homes with similar amount of residents was reported in a study from Schwaber et al., 2013 [9]. In this context, substantial MRSA transmission from residents to HCW’s gown and gloves has already been reported in residence homes, whereby high contact activities of daily living confer the highest risk [12]. Hospital based studies found colonization rates of HCW between 5.0 and 17.0%, a higher risk for colonization of HCW with frequent and intensive contact to patients [16, 27] and the contribution of HCW to the transmission of MRSA and subsequent infections [13, 15]. This leads to the question if MRSA transmission from colonized residents to nursing staff and from nursing staff to other residents could be a relevant route of transmission in residence homes. This question cannot be answered with the data from this study and needs to be evaluated in additional investigations.

In terms of organizational and technical feasibility, testing could be included in the residence homes avoiding any inconvenience to working time or negative impact on nursing quality. In similar studies, successful point-of-care testing for MRSA and other bacteria using real time PCR has already been demonstrated in hospital wards [36, 37]. During this study, it was essential to assign specimen collection and test procedures to employees with directive function in the institution who were able to adapt working time and working schedule to collect the specimen and perform the tests. This shows that such measurements in residence homes would need clear definition of responsibilities. Including all time-consuming steps, the average time needed for 1 testing procedure and 1 specimen collection procedure was 4 min and 18 s and 3 min and 27 s, respectively. Maximum time measured was 11 min for specimen collection and 9 min for MRSA testing. This low average time needed per specimen and test procedure was a tolerable investment within this study, in particularly in the period after baseline evaluation, where the frequency of specimen collection and testing of specimens was strongly reduced to about 1–2 per week. In terms of feasibility, 2 additional points needs to be considered. From a regulatory point of view, effective structures for external and internal quality control of such measurements in residence homes for the elderly needs to be established since these quality measures are mandatory in Germany [38, 39]. In addition, performing these quality control measures are a prerequisite for potential reimbursement of such measurements in Germany.

The study has some limitations. Firstly, the design and observational period of this study does not deliver a proof that the frequency of MRSA-transmission between residents and MRSA-infections of residents can be reduced via MRSA testing in residence homes for elderly. In a very similar study involving 4 resident homes with 2,492 residents, screening for MRSA and subsequent hygiene intervention did not result in a decrease in the prevalence of MRSA colonization within an observational period of 28 months [40]. Therefore, a large, prospective, population-based longitudinal study is required to define the adequate screening interval during the long term stay in the facility and adequate intervention methods, as recently reported [7]. In particular, the definition of adequate screening intervals are important because MRSA transmission in resident homes is documented [7, 8] and infections can appear shortly after acquisition. In a study conducted with adult hospitalized patients, the majority of patients developing clinical MRSA infection did so soon after acquisition, with 42.1% (51/121, 95% CI: 33.2 – 51.5) presenting with infection within 14 days of MRSA acquisition. This increased to 57.8% (70/121, 95% CI: 49.4 – 67.6) by 30 days and 71.9% (87/121, 95% CI: 63.0 – 79.7) at two months [41]. In addition there is evidence that risk factors for pre-existing colonization and new acquisition within nursing facilities can differ [8].

Secondly, it should be pointed out that during this study no measurements were performed in residents at the time of transfer from the facility to a hospital. It has been shown that cabin surfaces of ambulance cars transporting hospitalized patients are at risk of MRSA contamination and that cleaning procedures do not necessarily lead to complete eradication of bacteria. Even if the risk for acquisition during transportation in an ambulance is reported to be very low, it cannot be excluded [42, 43]. It needs to be taken into consideration that only nasal swabs have been investigated to define MRSA status during this study. Nasal swabs have the highest prediction for positive carrier status, but nevertheless MRSA colonization can take place also in other body sites like throat, perineum, intestine (stool) or hollow of the knee without colonization of the nose. Relating to this, involvement of extra-nasal testing body sites has been shown increased yield for MRSA colonization of up to 30.0% [44]. This potentially has led to an under-estimation of the true rate of colonized patients during this study and should be taken into account for further investigations.

## Conclusions

In this study, PCR-based screening for MRSA in nursing homes for elderly, followed by culture based confirmation in a laboratory, enabled the rapid detection of unknown carriers. As MRSA negative residents can acquire MRSA during a hospital stay, further spread after re-admission to the nursing home cannot be excluded. Attention should also be paid to nursing staff who can be carriers for MRSA and potential sources of transmission. Additional studies are needed to show if screening followed by interventional hygiene measures can reduce MRSA transmission and infection in such facilities. In Germany, a regulatory basis needs to be established respecting mandatory guidelines for quality control and reimbursement.

## Abbreviation

MSSA: Methicillin sensitive *Staphylococcus aureus* (S, aureus without genetic markers for resistance against Methicillin)
